# Supplementary material for: Facile Synthesis of Graphene from Waste Tire/Silica Hybrid Additives and Optimization Study for the Fabrication of Thermally Enhanced Cement Grouts
Source: Molecules. 2020 Feb 17;25(4):886. doi: 10.3390/molecules25040886 (PMC7070540; doi:10.3390/molecules25040886)
Supplement: Supplementary file 1 [file molecules-25-00886-s001.pdf]

Table S1. C/O ratios according to XPS results and

Samples	Carbon (%)	Oxygen (%)	C/O ratio
**GNP**	361644	144321	2.5
**Si:GNP=1:5**	220471	350888	0.6
**Si:GNP=1:10**	204405	249117	0.8

Table S2. Raman peak intensities, ID/IG ratios and crystallinity index of GNP, Si-GNP=1:5 and Si:GNP=1:10 hybrid additives

Samples	D peak intensity (a.u.)	G peak intensity (a.u.)	ID/IG	Crystallinity
**GNP**	2670.6	2754.5	0.97	24.1
**Si:GNP=1:5**	3043.9	3100	0.98	27.8
**Si:GNP=1:10**	3894.2	4111.6	0.94	23.3
